# Odorant Binding Proteins of the Red Imported Fire Ant, *Solenopsis invicta*: An Example of the Problems Facing the Analysis of Widely Divergent Proteins

**DOI:** 10.1371/journal.pone.0016289

**Published:** 2011-01-31

**Authors:** Dietrich Gotzek, Hugh M. Robertson, Yannick Wurm, DeWayne Shoemaker

**Affiliations:** 1 Department of Ecology and Evolution, University of Lausanne, Lausanne, Switzerland; 2 Department of Entomology, University of Illinois at Urbana-Champaign, Urbana, Illinois, United States of America; 3 Center for Medical, Agricultural, and Veterinary Entomology, United States Department of Agriculture – Agricultural Research Service, Gainesville, Florida, United States of America; Field Museum of Natural History, United States of America

## Abstract

We describe the odorant binding proteins (OBPs) of the red imported fire ant, *Solenopsis invicta*, obtained from analyses of an EST library and separate 454 sequencing runs of two normalized cDNA libraries. We identified a total of 18 putative functional OBPs in this ant. A third of the fire ant OBPs are orthologs to honey bee OBPs. Another third of the OBPs belong to a lineage-specific expansion, which is a common feature of insect OBP evolution. Like other OBPs, the different fire ant OBPs share little sequence similarity (∼20%), rendering evolutionary analyses difficult. We discuss the resulting problems with sequence alignment, phylogenetic analysis, and tests of selection. As previously suggested, our results underscore the importance for careful exploration of the sensitivity to the effects of alignment methods for data comprising widely divergent sequences.

## Introduction

Chemosensory systems play a central role in the way insects perceive their surroundings and are critical to finding mates, food, and oviposition sites. These olfactory and gustatory systems rely on at least two distinct protein families to translate environmental chemical signals into action potential. Since these proteins are thought to be the first interactants with the odorant semiochemicals they pose an important discriminatory filter during perception of chemosensory stimuli. Odorant binding proteins (OBPs) and chemosensory proteins (CSPs) are small, water-soluble, extracellular proteins, which bind hydrophobic semiochemicals in the lymphatic cavities of the sensory organs and transport them to the second class of proteins, the chemoreceptors [1]. Odorant binding proteins were first thought to have highly specific binding affinities to certain semiochemicals and to be exclusively expressed in the antennae of insects. However, both hypotheses have proven not to be correct. Although some OBPs appear to be exclusively involved in odor detection, others are expressed in various tissues and during different life stages (see [2] for a review), which suggests that the protein family can serve multiple functions. Whole genome surveys have shown that OBPs and CSPs are highly divergent protein families and are characterized by lineage-specific expansions, presumably driven largely by adaptation. To date, most insect genomes have been shown to contain around 40–55 OBPs and 4–8 CSPs [3]. The honey bee, *Apis mellifera*, is unusual in that it contains a low number of OBPs, only 21, and no significant expansion of CSPs [4]. Until recently [5], [6], no OBPs and only CSPs had been found in the antennae of ants, causing Calvello et al. [7] to speculate that functionally, the OBPs have been replaced by CSPs in these Hymenopterans. This hypothesis is consistent with the large number of CSPs in the red imported fire ant, *Solenopsis invicta* Buren, 1972, which possesses at least 14 CSPs [6]. However, the number of OBPs in this ant has not been determined.

For the present study, we attempted to identify and enumerate the full repertoire of OBPs in this ant. While such an endeavor previously was not feasible, the recent development of genomic resources for this ant now affords us with such an opportunity. The first such resource was an expressed sequence tag (EST) project in which >22,000 cDNAs were sequenced from both ends using Sanger termination methods, resulting in 21,715 ESTs representing 11,864 putatively different transcripts [7]. González et al. [6] recently described the chemosensory proteins (CSPs) revealed by the Sanger-based EST project, and here we describe the OBPs. The EST library [9] was augmented with data from two sequencing runs of massively parallel pyrosequencing using the Roche 454 FLX machine generating a total of 533,091 reads averaging 236 bp long and mined for sequences encoding OBPs.

To date, only one OBP has been described in detail from any ant, the locus *general protein-9* (*Gp-9*), which is implicated in regulating colony queen number in *S. invicta* and closely related fire ants [10], [11], [12], [13]. The *Gp-9* locus is unusual for an OBP in several ways – it displays high levels of variation in the coding region, is highly expressed, and found in the hemolymph of all castes [12], [13]. To provide the foundation for future studies of other fire ant OBPs, identification of all members of the OBP gene family in *S. invicta* is needed, and is the goal of the present study. We also use our data to further emphasize a general problem facing studies of widely divergent molecular sequences (which is one characteristic of insect OBPs) since the results obtained heavily depended on the underlying multiple sequence alignment method used, stemming no doubt from the large sequence divergence of these proteins. Our study highlight the necessity to carefully consider whether current analytical methods are adequate to analyze increasingly divergent molecular sequences (e.g., [14]) as well as the importance of investigating the influence of alignment methods on results.

## Results

### Identification of OBPs

The final assembly contains 18 contigs encoding *S. invicta* OBPs (SiOBPs), summarized in Table 1. One additional sequence similar to an OBP was also found (SiJWD04CAE), but it was so highly degenerate that it was not named and was dropped from all further analyses (this sequence shares the closest sequence identity with SiOBP3 [*Gp-9*]). Only a few of these contigs were full-length in the automated assembly, but manual re-assembly of the reads that belong to each contig allowed extension of 5′ and/or 3′ ends, generally yielding at least the entire coding sequence, and generally reaching a polyA tail. It is not possible to be confident that the 5′ ends of these contigs are the true transcription start site, so the cDNA lengths given in Table 1 are not necessarily definitive. It appears that the automated assembly was conservative in trimming reads for low quality ends, and in not extending contigs beyond apparent length differences in the constituent reads. Although most sequences derive from contigs comprising large numbers of 454 reads of ∼250 bases, eleven also have longer Sanger reads from the earlier published EST project [7], indeed three sequences are entirely from Sanger reads, with SiOBP18 being derived from a single Sanger read. Together with SiOBP17, these are also the two most problematic sequences. SiOBP17 appears to be partially unspliced with apparent intronic sequence interrupting the coding region, which is otherwise full-length, while the SiOBP18 read encodes only an internal part of this OBP, despite being quite long. The numbers of 454 reads contributing to each contig gives a rough estimate of their expression levels, with several clearly being well-expressed; SiOBP3, which has already been extensively studied as *Gp-9*, has an extremely large number of reads. The manual assembly of the 454 reads for several OBPs revealed that commonly more than one polyadenylation site was employed (listed in Table 1), and for those we employed the longest 3′ UTR available. Contig sequences encoding SiOBPs 1–16, excluding SiOBP3 which is already highly represented in GenBank as *Gp-9*, have been submitted to GenBank (HQ853350–HQ853364).

**Table 1:** Details of the *Solenopsis invicta* odorant binding proteins.

Gene	cDNA	TotAA	MatAA	454	Sanger	C	PolyA
SiOBP1	857	139	120	99	4	6	multi
SiOBP2	804	152	135	41	0	4	single
SiOBP3	631	153	134	>1200**	8	6	single
SiOBP4	638	153	134	14	0	6	none
SiOBP5	730	144	122	34	0	6	multi
SiOBP6	591	146	128	4	0	6	none
SiOBP7	623	133	116	335	2	6	single
SiOBP8	634	153	126	0	3	4	single
SiOBP9	859	129	109	21	0	6	multi
SiOBP10	747	147	131	43	1	6	single
SiOBP11	662	149	125	15	0	6	single
SiOBP12	936	174	154	35	4	6	multi
SiOBP13	740	160	144	14	9	6	single
SiOBP14	781	162	146	80	0	6	single
SiOBP15	894	162	140	112	9	6	multi
SiOBP16	660	171	155	40	4	6	multi
SiOBP17N*	834	168	148	0	1	6	single
SiOBP18N	654	>77	>77	0	1	6	none

*This single Sanger read appears to be partially unspliced and frameshifted.

**The total number of 454 reads contributing to this SiOBP3/*Gp-9* contig is unclear, because it strangely assembled in several different non-overlapping contigs.

The columns are: Gene – number we are assigning; cDNA – length of cDNA in base pairs, excluding polyA tail; TotAA – conceptual precursor protein length including signal sequence; MatAA – mature secreted protein length excluding signal sequence according to PSORTII; 454 – number of 454 reads contributing to contig; Sanger – number of Sanger reads contributing to contig; C – number of conserved cysteines; PolyA – presence of single or multiple poly-adenylation sites.

### Multiple sequence alignment

Due to the significant sequence divergence of the OBPs used in this study (overall ∼20% protein sequence identity), we were skeptical of the accuracy of any single multiple sequence alignment (MSA) to infer homologous amino acid residues of these divergent proteins. Hence, we compared six MSA methods, which employ widely different alignment methodologies and have been shown to perform well and/or are commonly used (Table 2). Additionally, we conducted simultaneous alignment and topology inference in a Bayesian framework using BAli-Phy for both the *Apis* and *Solenopsis* OBPs (AmOBPs; [4] and SiOBPs, respectively). Since this approach is generally considered to be conceptually superior to the generally used two phase methods, which separate alignment estimation and tree topology inference [15], [16], we considered the alignments and topologies derived from these searches to be the “true” tree.

**Table 2:** Details of the multiple sequence alignment (MSA) methods used and maximum likelihood phylogenies estimated from them.

rank	alignment	version	length	core length	LnL	parsimony	tree size	average aLRT	RF distance ant/bee	% seq.identity	reference
	BAli-Phy	2.0.2	253	130	−5736.1899	1229	14.24144		na	0.222	[51]
1	PRANK	1.0	332	152	***−10671.224***	***2284***	***28.84963***	0.86275	***4*** **/** ***2***	***0.223***	[67]
2	MUSCLE	3.6	206	117	−11160.1619	2520	34.76337	***0.877306***	***4***/4	0.203	[68]
3	MAFFT	6	209	115	−10900.89887	2448	34.152	0.866611	8/8	0.203	[69]
4	CLUSTALW	2.0.12	197	111	−10966.46551	2474	36.24757	0.843056	8/10	0.191	[70]
5	OPAL	1.0.3	219	127	−11178.67281	2511	37.78651	0.83475	8/10	0.203	[71]
6	SATCHMO	2.06	232	121	−11159.04352	2521	42.12012	0.792278	12/10	0.193	[72]

We define the core length as the number of character positions from the first to the last of the characteristic cysteine residues (C1–C6) of the OBPs. The log-likelihoods (LnL), parsimony informative characters, tree size, and average approximate likelihood-ratio tests (aLRT) are derived from the ML analyses. Robinson-Foulds tree distances (RF distance) are calculated by comparing the ant and bee MAP trees to the ML trees derived under the other MSA methods. Best scores of the MSAs compared to the BAli-Phy MAP are in highlighted in bold italics.

**Figure 1:**
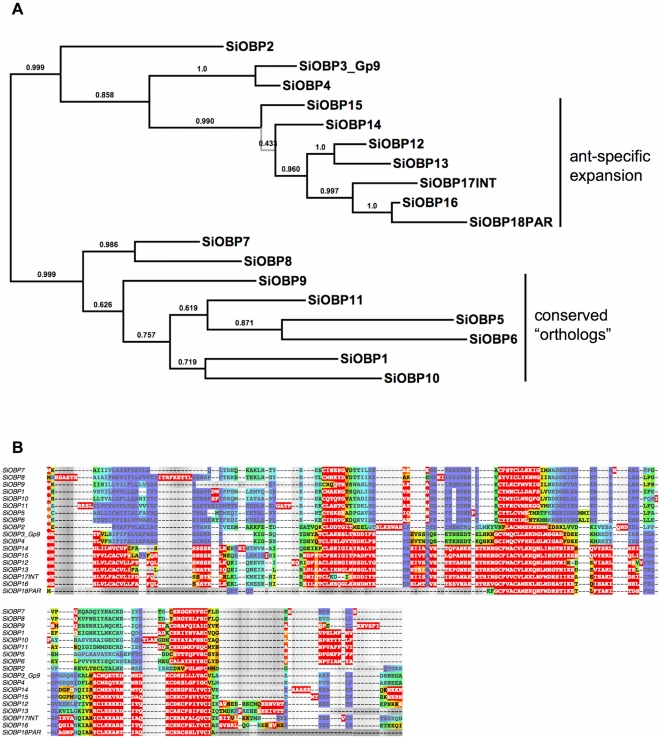
Maximum a-posteriori (MAP) phylogeny and alignment of the *Solenopsis invicta* (SiOBP) odorant binding proteins. A. The *S. invicta* MAP phylogeny. The branch in grey is collapsed in the 50% consensus tree. Branch support is posterior probabilities derived from 3241 samples taken after the burn-in was discarded. Even though the node support in the conserved ortholog clade is relatively poor, the exact same topology of the orthologs was recovered in the honey bee MAP tree (not shown), suggesting that the branching pattern is accurate. B. The *S. invicta* MAP-AU plot. The quality of the alignment is indicated through a heat map. Red (warm colors) indicates areas of high quality alignment, blue (cold colors) signifies areas of low certainty. Note that there are considerable high quality alignment blocks in the N-terminal signal peptide and the C-terminal protein tail.

It is common practice to account for the wide divergence between OBPs by removing signal peptides and less often the C-terminal residues prior to multiple sequence alignment and, hence, to restrict the following analyses to the presumed more conserved “core” of the proteins [e.g.], [ 17], [18], [19,20]. However, Wong *et al.* [14] advise against eliminating difficult blocks from alignments, since some of these may still contain informative sites and their removal does not necessarily result in more concordant inferences. Additionally, they show that it is possible to make inferences despite considerable alignment uncertainty. Hence we did not remove areas of uncertain alignment, especially since the AU plots of both the *Solenopsis* and *Apis* BAli-Phy alignments suggest that there are still high quality alignment blocks within these “problematic” areas to warrant their inclusion in the overall alignment procedure (Figure 1b). This is especially true for the signal peptides, which are most often removed before analyses [17], [18], [19], [20]. Moreover, we do not consider the “core” sequences to be inherently more informative than the outside areas, since the lengths of the core (which we define as ranging from C1 to C6) differed greatly between MSA methods (Table 2). Preliminary analyses also suggest that removing the outer areas do not change significantly the topology derived from them (data not shown). Additionally, both the Steel [21] and Xia [22] tests indicated high levels of sequence saturation for our dataset for all MSAs (not shown), suggesting that the dataset contains little useful evolutionary signal.

### Phylogenetic analyses

**Figure 2:**
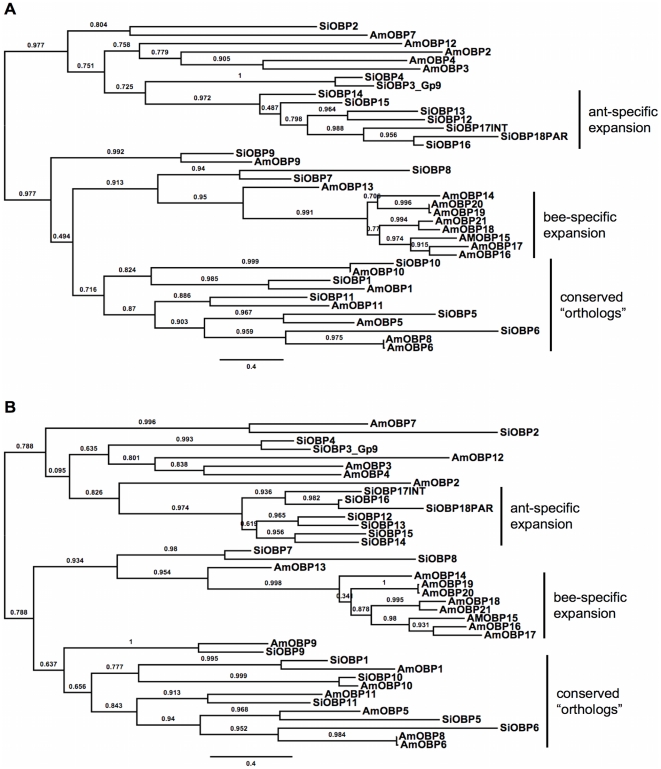
Maximum likelihood phylogenies of the fire ant OBPs (SiOBPs) and honey bee OBPs (AmOBPs). The phylogenies are based on the two best alignments (top: MUSCLE, bottom: PRANK). All trees are midpoint rooted in the absence of a suitable outgroup. Branch support is SH-like aLRT derived from PhyML analyses.

Despite the great difference in alignment lengths and the pronounced sequence saturation as shown by the Steel and Xia tests, most MSAs still yielded highly similar tree topologies. Several clades were consistently recovered and the midpoint root was generally placed in the same position across all MSAs (Figure 1a, Figure 2). So despite the obvious problems to align the widely divergent OBP dataset, we conclude there is enough phylogenetic information in the alignments to at least draw tentative conclusions regarding the evolution of fire ant OBPs. The maximum likelihood and two Bayesian searches recovered highly similar tree topologies, with the Bayesian trees generally being less resolved, especially at the deeper nodes.

### Selection analyses

Forêt and Maleszka [4] described evidence of positive selection in the AmOBP expansion, so we used estimates of dN/dS (ω) to examine whether the same was true of the SiOBP expansion. Given the uncertainties of alignment and topology, we conducted site-specific tests of selection [23], [24], [25], [26] on the two best (PRANK, MUSCLE), the shortest (CLUSTAL), and the *Solenopsis* MAP alignments (Table 3). Site specific analyses of all OBPs combined showed no evidence of positive selection for either the PRANK or MUSCLE alignments. The ant MAP alignment, however, showed a signature of positive selection using the M1a (neutral)–M2a (selection) comparison, but not the M7–M8 comparison, which has been shown to be less robust (but more powerful) than the M1a–M2a comparison [23]. For the M1a–M2a comparison, the Bayes Empirical Bayes (BEB) method identified two amino acid positions in the core (aa81 with PP = 0.991 and aa128 with PP = 0.979) as being under positive selection (ω = 2.9485). Even though the M7–M8 comparison was not significant, the BEB indicated the same sites (aa81 and aa128) have elevated ω estimates (ω = 1.8266). The CLUSTAL alignment contained evidence of positive selection for both tests (M1a–M2a: ω = 2.0973, aa20 PP = 0.972, aa25 PP = 0.981, aa49 PP = 0.981, aa70 PP = 0.955, aa133 PP0.995, aa177 PP 0.976, aa178 PP = 0.999; M7–M8: ω = 3.5508, aa178 PP = 0.964). The two amino acid positions in the core of the CLUSTAL alignment identified to be under positive selection (aa70 and aa133) are not identical to those of the MAP alignment, suggesting that the tests of positive selection using different alignments are not picking up the same evolutionary signals.

**Table 3:** Results of the selection analyses for the best alignment method (PRANK), and two others (CLUSTAL and MUSCLE) and the MAP dataset of *Solenopsis*.

Site model						
	M1a	M2a	LRT	M7	M8	LRT
CLUSTAL	−16240.8767	−16219.8	42.1534***	−16048.4291	−16046.0607	4.7369***
MUSCLE	−16330.4985	−16330.3	0.4105	−16270.4	−16269.6	1.6646
PRANK	−15838.686	−15838.7	0	−15772.4	−15772.4	0
MAP	−8454.7161	−8446.69	16.0498***	−8399.03	−8398.04	1.9778

Branch model			
	H_0_	H_a_	LRT
CLUSTAL	−16184.9809	−16183.7734	2.4152
MUSCLE	−16478.8518	−16474.628	8.4477***
PRANK	−15891.3104	−15886.5809	9.4588***
MAP	−8453.96731	−8452.55841	2.8178

Branch-site model			
	H_0_	H_a_	LRT
CLUSTAL	−16112.0983	−16111.4054	1.3857
MUSCLE	−16334.5808	−16333.4641	2.2334
PRANK	−15842.8377	−15841.8544	1.9667
MAP	−8424.3691	−8424.3426	0.0529

Given are the log-likelihoods of the null hypotheses (H_0_), which assume no selection, and alternative hypotheses (H_a_), which allow for positive selection. Likelihood-ratio tests (LRT) of positive selection are conducted to compare the two hypotheses. Levels of significance are 3.84 at 5% and 6.63 at 1% for the site model and 3.84 at 5% and 5.99 at 1% for the branch and branch-site models, following the χ_1_^2^ distribution to guide against violations of model assumptions. Statistically significant LRTs for positive selection are indicated by italics and *** for p≪0.01. Note that inference of positive selection greatly depends on the alignment method used.

We tested whether these signatures of positive selection were associated with the ant-specific expansion, which we tested using branch-specific tests of selection [27], [28]. Oddly enough, the LRT comparing the null and alternative hypotheses showed significant differences in the PRANK and MUSCLE MSAs, suggesting episodes of positive selection on this branch. However, in both cases the estimates of ω for this branch were <1 and even lower than the estimate of ω across all other branches. This pattern is consistent with relaxed selection, especially since it is coupled with a rapid gene expansion in this clade. The explanation of increased purifying selection to explain this pattern seems less likely to us. However, the branch-specific test for selection averages the estimates of ω across the whole sequence length and as a result may lack power [29] and obscure episodes of positive selection restricted to one or very few sites. Hence, we also applied branch-site analyses of selection [26], [30] on the branch leading to the ant-specific expansion. These tests were not significant for any of the datasets, supporting our interpretation of lack of positive selection.

## Discussion

We identified a total of 19 OBPs in *S. invicta*, of which 18 appear to be putatively functional. The red imported fire ant thus appears to possess a small set of OBPs similar to that of the honey bee *Apis mellifera* (21 OBPs [4]). Although this estimate may slightly change with the assembly and annotation of the complete fire ant genome [31], the fire ant OBP repertoire is one of the smallest reported among insects, with only the pea aphid *Acyrthosiphon pisum* and the body louse, *Pediculus humanus*, appearing to have fewer OBPs (15 and 5, respectively; [20], [32]). Preliminary scans of the coding regions (CDS) and peptide libraries of the jumping ant, *Harpegnathos saltator*, and the carpenter ant, *Camponotus floridanus*, genomes (both version 3.3 [33]) found twelve and seven OBPs respectively. While additional annotation efforts on these genomes likely will increase the number of OBPs to comparable levels of *Solenopsis* and *Apis*, it does appear that the social Hymenoptera in general possess relatively few OBPs. Ongoing and future genome projects in other bees and ants will prove important to address this issue.

### Multiple sequence alignment

The MSAs resulted in alignments of widely different lengths and quality (Table 2). The best alignment method, as measured by the Robinson-Foulds distance to the BAli-Phy topologies, was PRANK followed by MUSCLE. These two methods also produced the “best” fitting trees to the data by any measurement (LnL, tree length, branch support). The other MSAs (MAFFT, CLUSTAL, OPAL, SATCHMO) fared worse and were never “best” by any measure. MAFFT, however, came in second to PRANK in the estimates of LnL, parsimony, tree length, branch support, and percent sequence identity. The quality of the alignments do not seem to be contingent upon the total lengths or the core lengths, since PRANK is by far the longest alignment and MUSCLE is the second shortest. Additionally, both the Steel [21] and Xia [22] tests indicated high levels of sequence saturation for our dataset for all MSAs (not shown), suggesting that the OBP alignments contained little evolutionary signal.

Also, the AU plots (Figure 1b) suggest that the common removal of signal peptides [17], [18], [19], [20] may not be necessary, since these areas still possess high quality alignment blocks.

### Phylogenetic analyses

The phylogenetic relationships of the 18 functional fire ant OBPs (SiOBPs) to the 21 OBPs described from the honey bee, *Apis mellifera* (AmOBPs [4]) are shown in Figure 2. We named the SiOBPs in a numerical series attempting as best possible to use the same numbers for those showing high conservation and presumed orthology with a subset of the honey bee OBPs (Figure 2). These are OBPs 1, 5, 6, 9, 10, and 11 (AmOBP6 and 8 are almost identical in encoded amino acid sequence, but derived from adjacent slightly different genes). Our phylogenetic assessment of orthology in these OBPs is robust across all alignment methods (despite moderate branch support in some cases), suggesting that the assignment is accurate. The phylogenetic analysis indicates that these conserved orthologs may constitute a monophyletic lineage, albeit without high branch support. Even though not all MSAs recovered the same relationship among these orthologs, nor their monophyly, the *Solenopsis* and *Apis* BAli-Phy trees share identical branching patterns for these orthologs, suggesting that the phylogenetic information within these sequences was conserved during cladogenesis. This branching pattern was also recovered by the PRANK alignment method, even though the branch support for the deeper nodes is relatively poor.

Phylogenetic analyses also suggest a close relationship between these same orthologs and a bee-specific clade (AmOBPs 14–21), which is comprised of OBPs encoded by a tandem array that are distinct in having lost a pair of the six usually conserved cysteines (so-called “C-minus” OBPs) and also exhibiting signals of positive selection [4]. Although AmOBP13 is also in this tandem array, this OBP has six cysteines and is not expressed in adult antennae but rather in late larval and early pupal stages [4]. SiOBP7 and SiOBP8 are sister to the C-minus expansion and AmOBP13, but with weak support. SiOBP8 has lost the same pair of cysteines (Table 1), apparently independently of the losses in the honey bee, which in turn are independent of other losses of this pair of cysteines in other C-minus OBPs in other insects [4].

The other half of the tree contains another mixture of AmOBP and SiOBP lineages. SiOBP2 has lost the same pair of cysteines as SiOBP8, and this loss also seems to be independently derived, since it always clusters with AmOBP7 (except in the SATCHMO alignment, not shown) with modest branch support. AmOBPs 2–4, and 12 cluster together with weak support. SiOBP3 is *GP-9*, the OBP implicated in control of social behavior in these ants [10], [12], and SiOBP4 apparently is a paralog: These proteins share only 68% amino acid identity, but are co-linear. SiOBP4 is 87% identical to a supposed divergent ortholog of *GP-9* from an unidentified “thief ant” species (GenBank AAW80681 [34]). This suggests that the supposed thief ant *GP-9* is more likely an ortholog of SiOBP4 and that *GP-9* may be restricted to the fire ants (*geminata* species group [35], [36]). While these proteins have no consistent relationship to any of the honey bee OBPs, SiOBP3 and SiOBP4 are the sister group to a seven-gene ant-specific OBP expansion (SiOBP12–18), which itself is close in size and in rate of radiation to the C-minus AmOBP14–21 gene expansion.

It is tempting to speculate that like the OBPs of the bee-specific expansion, these relatively young ant-specific OBPs might well constitute a major fraction of those expressed in the antennae and thus may serve as part of the primary olfactory OBPs in *S. invicta*. However, whether any of these proteins are directly involved in olfaction remains to be demonstrated. Circumstantial evidence suggests that this is unlikely. In fact, the use of OBPs in ant chemosensation has been questioned. Previous studies were unable to identify any members of this protein family in ant antennae [37], [38], [39], which led Calvello *et al.* [7] to speculate that ants may prefer to use CSPs instead of OBPs for olfaction, which could explain the expansion of CSPs in *S. invicta*. More recently however, three OBPs have been documented [5], [6, R. Renthal personal communication] in the antennae of red imported fire ant workers (SiOBP15 [OBP1 of Wang *et al.* [7]], SiOBP3 [*GP-9*], and SiOBP2). None of these proteins appear to be orthologous to any AmOBPs, which have been shown to be expressed in the bee antennae. While the bee OBP data suggest that expression in antennae (and the concomitant presumed use in chemosensation) is phylogenetically preserved, this view may well be biased because half of the AmOBPs tested belong to the rapid bee specific expansion [4].

### Selection analyses

The varied and mixed results of the selection analyses suggest that any selection analyses of OBPs be viewed with healthy skepticism. As Wong *et al.* [14] demonstrated, alignment variability is positively and significantly correlated with the number of non-synonymous substitutions, which could explain our positive results for the site- and branch-specific tests of selection and those of Forêt and Maleszka [4]. More recently, Fletcher and Yang [40] showed that alignment errors can lead to a high number of false positives for the branch-site test of positive selection. Even the best performing MSA method (PRANK) did not have the false-positives under control, but nonetheless did fare better than the other alignment methods (MAFFT, MUSCLE, and CLUSTAL) [40]. However, our branch-site tests of selection did not reveal any evidence of positive selection on the branch leading to the ant-specific expansion for any of the alignments used, suggesting that alignment error may not have been an important issue for these analyses. Thus, we are left in the unfortunate position of not being able to conclude confidently the nature of selective forces, if any, shaping the evolution of OBPs in *S. invicta* (and the honey bee), except to say that, like in other insects, lineage-specific expansions are a common feature of Hymenopteran OBP evolution and that their OBPs are widely divergent.

Perhaps more importantly, our data suggest that inferences drawn from analyses of widely divergent molecular sequences are to be regarded with skepticism, since the outcome heavily depends on the resulting alignment chosen. While these issues have been raised previously [e.g.], [ 14], [40], [41], [42], [43], [44,45], such analyses are becoming increasingly commonplace, especially with the advent of next-generation DNA sequencing platforms and the rapid increase in genomic data, yet, many researchers appear not to consider the estimation of molecular sequence alignment as an exploratory phase of data analysis [46]. Rather, the inference of tree topology is explored much more often, where the judicious choice and use of underlying models, optimality criteria, branch support measures, etc. are a mandatory consideration in virtually all publications and the potentially different outcomes are discussed critically. This apparent lack of attention to MSA methods perhaps stems from an era when the study of molecular sequences was limited to what could be successfully amplified, which likely led to biased analyses of closely related sequences. In any case, we concur with earlier studies that there is an increasing need for awareness for the necessity of careful and critical data exploration during all stages of molecular evolutionary analyses [14], [44], [46].

## Materials and Methods

### Identification of loci

Odorant binding proteins and chemoreceptors were identified using BLAST searches [47] of the combined EST and preliminary 454 sequencing data using the fruit fly [48] and honey bee OBPs [4]) as query. The fire ant genes thus identified were then iteratively used as BLAST queries against the same fire ant sequence database until no further new *Solenopsis* loci were found. After we had concluded all our analyses, we also used BLAST searches against the predicted proteins and CDS of the recently released *Camponotus floridanus* and *Harpegnathos saltator* genomes v. 3.3 [33] using the *Apis* and *Solenopsis* OBP amino acid sequences as queries. Given the incomplete annotation of the genomes and the low number of OBPs recovered, we chose not to perform analyses including the other ant OBPs, but instead defer to future researchers that can make use of the several other ant genomes currently being sequenced to address this issue more fully [31].

### Multiple sequence alignment

Expecting the generally divergent nature of OBPs sequences (∼20% amino acid identity over all sequences) to make the sequence alignment problematic [49], we used several multiple sequence alignment (MSA) methods to evaluate potential different outcomes of using six alignment approaches (Table 2), which differ greatly in popularity and general approach to the MSA problem [50]. We used default parameters for all alignment estimates. Nucleotide (codon) alignments were based on the amino acid alignments.

In addition, we used BAli-Phy 2.0.2 [51] to simultaneously estimate the alignment and phylogeny of the each species' OBPs in a Bayesian framework [52]. Since BAli-Phy is computationally intensive and generally considered to be too slow to be efficiently used with more than a dozen sequences, we conducted these analyses for both the ant and bee datasets independently. Additionally, we removed six bee OBPs from the well-supported C-minus expansion [4] to reduce computational burden. We used default parameters for each run of 100,000 generations. Stationarity of the searches was verified using Tracer 1.5 [53]. 9999 samples were removed in the burn-in. The lowest effective sample size (ESS) for any parameter estimate was 802.3378, suggesting that we had run the analyses sufficiently long to enable meaningful estimates from the posterior sampling.

The alignments were compared using a range of ad hoc heuristic criteria. First, we visually compared alignments for congruence in their ability to align sections of the alignments (especially the inner core) using AltAVisT [54] and the overall sequence identity calculated from each alignment. We then tested for sequence saturation using both the Steel (for amino acids; [21]) and the Xia (for nucleotides [22]) methods [55] using DAMBE [56]. Finally we compared their ability to capture phylogenetic signal relative to the other alignment methods (using ML trees; see below). To this end, we compared log-likelihoods, tree length (measured by parsimony steps of the phylogeny and ML tree size), and the average of aLRT branch support [57] as well as the Robinson-Foulds tree distance [58] to the ant and bee MAP trees using the TreeDist program in the PHYLIP 3.69 package [59].

### Phylogenetic analyses

We used the ProtTest server [60] to estimate the best-fitting model of amino acid substitution for each alignment using the Bayesian information criterion (BIC [61]). Tree topologies were optimized starting from an initial BioNJ tree. Phylogenetic hypotheses under the maximum likelihood criterion were derived from the amino acid alignments using PhyML3 [62]. We implemented the model consistently chosen by the BIC (LG [63]) while estimating the proportion of invariable sites (+I) and gamma shape parameter (+Γ) with 4 rate categories. Tree searches started from five random starting trees and used SPR and NNI to optimize topologies. Branch lengths were optimized and branch support was estimated using the SH-like aLRT [57]. We also employed MrBayes 3.1.2 [64] to compare phylogenetic hypotheses derived from the amino acid and nucleotide datasets. Due to computational burden of the Bayesian analyses, we only performed these on the two best alignments (MUSCLE and PRANK). For each alignment, we performed two searches using different models of sequence evolution. For the amino acid dataset we employed model averaging [65] to incorporate model selection in the Markov Chain Monte Carlo (MCMC) search. For the nucleotide codon alignment we implemented the GTR+I+Γ model. Four chains were run for 5 million generations (one cold and three heated; temperature = 0.02–0.03). Samples from the MCMC were taken every 1000^th^ generation. All other parameters were left at program defaults. Convergence was assessed by measuring average standard deviations of split frequencies, potential scale reduction factor (PSRF) values, plateauing of log-likelihoods values, and ESS values >100.

### Selection analyses

We conducted analyses of positive selection using the codeml program in the PAML 4.3 package [66]. Since codeml requires a fully resolved tree, we used the ML trees of the PRANK, MUSCLE, CLUSTAL, and BAli-Phy alignments as input. These represent the two “best”, the longest and shortest alignments. We estimated branch lengths under the F3×4 codon model on the respective topologies. We conducted site-specific tests of selection [23], [24], [25], [26]. We were also specifically interested in whether positive selection had influenced the divergence of the ant-specific expansion. Hence, we performed branch-specific tests of selection [27], [28] on the branch leading to this clade. However, under certain circumstances the branch-specific test of selection can lack power and so we also used the branch-site test of selection [26], [30] implementing the Bayes empirical Bayes (BEB [26]) method to identify sites under selection. To ensure that the analyses had converged properly, we repeated each analysis three times from different starting parameter options and under different codon models.
